# Gene-Network Analysis Identifies Susceptibility Genes Related to Glycobiology in Autism

**DOI:** 10.1371/journal.pone.0005324

**Published:** 2009-05-28

**Authors:** Bert van der Zwaag, Lude Franke, Martin Poot, Ron Hochstenbach, Henk A. Spierenburg, Jacob A. S. Vorstman, Emma van Daalen, Maretha V. de Jonge, Nienke E. Verbeek, Eva H. Brilstra, Ruben van 't Slot, Roel A. Ophoff, Michael A. van Es, Hylke M. Blauw, Jan H. Veldink, Jacobine E. Buizer-Voskamp, Frits A. Beemer, Leonard H. van den Berg, Cisca Wijmenga, Hans Kristian Ploos van Amstel, Herman van Engeland, J. Peter H. Burbach, Wouter G. Staal

**Affiliations:** 1 Department of Neuroscience and Pharmacology, Rudolf Magnus Institute of Neuroscience, University Medical Center Utrecht, Utrecht, the Netherlands; 2 Complex Genetics Section, Department of Biomedical Genetics, University Medical Center Utrecht, Utrecht, the Netherlands; 3 Department of Medical Genetics, University Medical Center Utrecht, Utrecht, the Netherlands; 4 Department of Child and Adolescent Psychiatry, Rudolf Magnus Institute of Neuroscience, University Medical Center Utrecht, Utrecht, the Netherlands; 5 Department of Neurology, Rudolf Magnus Institute of Neuroscience, University Medical Center Utrecht, Utrecht, the Netherlands; 6 Department of Psychiatry, Rudolf Magnus Institute of Neuroscience, University Medical Center Utrecht, Utrecht, the Netherlands; 7 Department of Genetics, University Medical Center Groningen, and University of Groningen, Groningen, the Netherlands; University of Muenster, Germany

## Abstract

The recent identification of copy-number variation in the human genome has opened up new avenues for the discovery of positional candidate genes underlying complex genetic disorders, especially in the field of psychiatric disease. One major challenge that remains is pinpointing the susceptibility genes in the multitude of disease-associated loci. This challenge may be tackled by reconstruction of functional gene-networks from the genes residing in these loci. We applied this approach to autism spectrum disorder (ASD), and identified the copy-number changes in the DNA of 105 ASD patients and 267 healthy individuals with Illumina Humanhap300 Beadchips. Subsequently, we used a human reconstructed gene-network, Prioritizer, to rank candidate genes in the segmental gains and losses in our autism cohort. This analysis highlighted several candidate genes already known to be mutated in cognitive and neuropsychiatric disorders, including *RAI1*, *BRD1*, and *LARGE*. In addition, the *LARGE* gene was part of a sub-network of seven genes functioning in glycobiology, present in seven copy-number changes specifically identified in autism patients with limited co-morbidity. Three of these seven copy-number changes were *de novo* in the patients. In autism patients with a complex phenotype and healthy controls no such sub-network was identified. An independent systematic analysis of 13 published autism susceptibility loci supports the involvement of genes related to glycobiology as we also identified the same or similar genes from those loci. Our findings suggest that the occurrence of genomic gains and losses of genes associated with glycobiology are important contributors to the development of ASD.

## Introduction

Autism Spectrum Disorders (ASD) consist of three disorders; autism, Asperger syndrome, and pervasive developmental disorder-not otherwise specified. These disorders can be distinguished according to symptom severity and symptom pattern, and by age of onset of the first symptoms. ASD are characterized by three core features: impairment in reciprocal social interactions, communicative deficits, and repetitive and restricted patterns of behavior and interests (Diagnostic and Statistical Manual of Mental Disorders-fourth edition-text revision, DSM-IV-TR). Individuals with impairments in all three areas and an age of onset before 36 months are diagnosed with autism. The risk for autism is mainly determined by genetic factors, as is the case for many psychiatric disorders, with heritability estimates for autism as high as 90% [1], [2]. Despite the evidence for major genetic contributions, the isolation of specific risk genes for autism has proven difficult, and in only a minority of autism cases a genetic defect can be unequivocally linked to the disease. Correspondingly, replication of linkage and association findings in autism cohorts has been problematic. The limited rate of reproducible findings may reflect extensive heterogeneity and modest effects of disease genes, effects of parental imprinting, and epistasis but also heterogeneity across populations, false-positive results, systematic bias due to technical artifacts, population stratification or environmental modifiers [3]. Alternatively, the involvement of rare variants may by definition be difficult to replicate across different patient cohorts.

Cytogenetic analysis has demonstrated a relatively high frequency of microscopically visible chromosomal abnormalities (3–5%) in individuals with ASD [4]. Fluorescence in situ hybridization (FISH) and even more powerful detection methods such as microarray-based comparative genomic hybridization (array-CGH) have demonstrated that submicroscopic genomic losses and gains may be causally related to autism at much higher percentages [5], [6]. These genomic gains and losses are often referred to as copy-number variants (CNVs), and can be transmitted by or arise *de novo* on both the paternal or maternal inherited chromosomes [7]. Recently, we have reported on clustering of autism-associated cytogenetic abnormalities in a large sample of independent case reports [4], and several others have reported the occurrence of multiple autism-associated CNVs [5], [6], [8], [9]. However, the identified loci are often large, covering many genes, and are often “private” to an individual or family. The challenge will be how to identify the causative dosage-dependent gene(s) within these regions. While the involvement of single gene mutations in individual autism cases cannot be excluded, the concept of a complex genetic model with multiple genes contributing to disease susceptibility remains highly plausible for the majority of cases [10]. In this context it appears to be justified to shift from a narrow focus on individual candidate genes towards a broader view of affected gene networks and associated biological pathways.

One approach towards the identification of such networks involves the integration of available biological data on individual genes, relating to their expression profile and the functional and structural properties of the encoded proteins. Based on this concept we have recently described a novel method for ranking candidate genes within disease susceptibility loci based on proven and/or putative interactions with genes present in other loci linked to the same disorder: Prioritizer [11]. The basic premise of this method is that the many susceptibility genes involved in a complex disease are mostly confined to a limited number of different biological systems and processes and therefore are likely to cluster within a reconstructed functional human gene-network. By using Prioritizer biological relationships between otherwise seemingly unrelated loci can be revealed, improving the odds for selecting the relevant candidate genes. Several other groups have since reported slightly different bioinformatics platforms for candidate gene prioritization, e.g. Endeavour, SUSPECTS, and ToppGene [12]–[14]. However, these methods use training sets of known disease genes and sequence similarity analysis that biases the analysis towards a limited number of known disease genes. An important strength of Prioritizer is that it functions without any a priori knowledge, e.g. which gene or protein sequence, organ, functional system, or diagnosis to look at, making it a less biased approach compared to other methods.

Here we report the results from the application of Prioritizer to CNVs identified in a microarray-based genome-wide CNV analysis in a phenotypically well-defined cohort of 105 Dutch ASD patients and 267 ethnically-matched healthy individuals, and on 13 additional autism susceptibility loci reported in the literature (including linkage regions and regions with recurrent genomic gains and losses). Comparison of these datasets suggests that genes involved in glycobiology are involved in the development of autism.

## Results

### Genome-wide CNV analysis using Illumina HumanHap300 Beadchips

We SNP-genotyped the DNA of 105 autism patients and 267 unrelated healthy controls using Illumina HumanHap300 Beadchips and Illumina Beadstudio analysis software (see methods for details). The autism cohort was divided into two patient groups according to the presence of a positive family history, growth disorders, and dysmorphic features or congenital anomalies. The first group consisted of patients with little or none of these abnormalities in concurrence with autism (non-complex-autism group, n = 53). The second group consisted of patients with more of the aforementioned abnormalities in concurrence with autism (complex-autism group, n = 52). The data obtained were subsequently analyzed for CNV content using a script previously shown to yield highly reproducible and reliable results [15]. In total, 452 CNVs were identified in the autism subjects and 1,433 CNVs were identified in the healthy controls. Table 1 summarizes the characteristics of these CNVs. 112 out of the 210 CNVs in the non-complex-autism group (53%), and 106 out of the 242 CNVs in the complex-autism group (48%) were not encountered in our control cohort. The mean number of CNVs detected per individual, irrespective of the type of aberration, did not significantly differ between the non-complex-autism group and complex-autism groups, or between the autism patient groups and control cohort (Wilcoxon-Mann-Whitney (WMW) test, p>0.05). The number of gains and losses in the non-complex-autism group was significantly lower than observed in healthy control individuals (WMW test, p-values for gains and losses respectively 0.031, and 0.001). In the complex-autism group, a nominally higher number of homozygous losses was present (WMW, p = 0.026). The mean size of the CNVs did not differ significantly between the autism groups and healthy control group, when excluding a 12.47 megabase segmental gain spanning the centromere, identified in a single patient in the non-complex-autism group (WMW test, p>0.05).

**Table 1:** Characteristics of CNVs identified in 105 autism patients and 267 healthy controls.

	Non-complex-autism	Complex-autism	Controls
	(n = 53)	(n = 52)	(n = 267)
**CNV totals** (average/individual)	210 (3.96)	242 (4.57)	1,433 (5.37)
**Duplications**			
N	97	109	648
Mean per individual (range)	1.83 (0–5)	2.10 (0–6)	2.44 (0–10)
Mean size in kilobases (range)	327.5 (4.3–12,478.8)	170.8 (3.4–2,881.4)	143.3 (3.9–9,583.7)
**Heterozygous deletions**			
N	109	124	771
Mean per individual (range)	2.06 (0–7)	2.38 (0–8)	2.90 (0–9)
Mean size in kilobases (range)	124.3 (2.4–554.9)	107.7 (0.8–3,043.6)	70.6 (1.0–1,348.6)
**Homozygous deletions**			
N	4	9	14
Mean per individual (range)	0.08 (0–1)	0.17 (0–2)	0.05 (0–1)
Mean size in kilobases (range)	42.7 (6.3–93.6)	46.9 (1.1–162.2)	82.1 (4.8–190.3)

### Prioritizer analysis of CNVs identified in autism patients

Subsequently, we attempted to select candidate genes and uncover shared biological processes by Prioritizer gene-network analysis, with solely the identified CNV regions as the point of departure. The identified CNVs were combined if they overlapped, resulting in 173 non-overlapping unique copy-number variant regions (CNVR), containing 347 genes, for the non-complex-autism group (Table S1), and 181 non-overlapping unique CNVR, containing 221 genes, for the complex-autism group (Table S2). Analyses were compared to 1,433 CNV regions that had been identified in the 267 healthy controls. If CNVs in different healthy controls overlapped, these CNVs regions were concatenated, resulting in 869 non-overlapping unique CNVR (containing 957 genes). We used this control dataset to control for the fact that many CNVs are common in the population. Additionally we used this control dataset to prevent for potential biases due to the non-uniform genomic coverage of the oligonucleotide array that had been used, resulting in more conservative, yet more robust results (see Materials & Methods for details). To eliminate biases towards genes that have many known or predicted interactions, a so-called topology-corrected analysis was performed (see Materials & Methods). Tables 2 and 3 show the topology-corrected significant results of Prioritizer analysis of the genes in CNVs found in the non-complex-autism and complex-autism groups, respectively. The non-complex-autism group showed an enrichment in the CNVs for interacting genes, given the fact that the 38 genes for which Prioritizer calculated p-values <0.05 are substantially more then the 17 genes expected by chance alone (0.05*347 genes). No such enrichment was observed in CNVs of the complex-autism group, as only 5 genes had a p-value <0.05 whereas by chance alone 11 genes could be expected with this score (0.05*221 genes).

**Table 2:** Prioritizer analysis of non-complex-autism patient CNVs.

Gene Name	Chr	Gene start	Gene end	P-Value
RAI1	17	17,525,512	17,657,400	0.001199
S100A5	1	150,322,696	150,327,314	0.001999
SDF4	1	1,180,538	1,207,334	0.003998
C14orf147	14	33,971,905	34,001,313	0.004398
KHDRBS2	6	62,447,824	63,054,091	0.004798
ZBED4	22	48,568,390	48,602,951	0.007596
NTM	11	131,286,586	131,710,250	0.009196
ORAI3	16	30,867,916	30,873,757	0.009196
**LARGE**	22	31,993,401	32,640,964	0.009196
PCLO	7	82,223,443	82,436,559	0.010795
DUOXA1	15	43,196,864	43,209,367	0.011195
DPF1	19	43,394,182	43,420,994	0.012794
SETD1A	16	30,876,116	30,903,482	0.014394
ATMIN	16	79,627,142	79,636,280	0.015193
LILRB3	19	59,412,549	59,438,461	0.016393
**B3GALT6**	1	1,207,568	1,210,341	0.017992
GCSH	16	79,673,430	79,687,481	0.017992
LOC729920	7	16,028,931	16,234,010	0.019992
LILRA3	19	59,491,671	59,496,077	0.021591
BRD1	22	48,487,793	48,541,685	0.023990
ZC3H7B	22	40,022,067	40,080,651	0.025589
CHMP5	9	33,255,000	33,322,656	0.026789
NANOGP1	12	7,916,801	7,943,941	0.027988
PPP1R14A	19	43,433,725	43,439,012	0.027988
TFAP2D	6	50,789,216	50,848,705	0.030387
HMGB1	3	22,398,314	22,572,730	0.031587
NOC4L	12	131,295,223	131,303,216	0.032786
DMRTC2	19	47,040,864	47,047,604	0.034386
TEF	22	40,102,463	40,119,828	0.034386
YIF1B	19	43,487,593	43,498,400	0.035985
FLJ16220	4	132,632,497	132,702,567	0.037584
FAM128A	2	132,075,266	132,083,834	0.037984
MLL5	7	104,248,588	104,348,759	0.038384
PARP8	5	49,998,570	50,173,926	0.040783
**GCNT2**	6	10,663,935	10,737,586	0.043182
IL17A	6	52,159,144	52,163,395	0.043582
ZNF208	19	20,519,337	20,599,769	0.047580
**GALNT9**	12	131,347,882	131,516,139	0.048780

The 38 most significant genes (nominal empiric P-Value<0.05) identified by topology-corrected Prioritizer analysis of 173 non-overlapping CNV regions are given. Gene start and end positions based on NCBI V35 assembly. Genes related to glycobiology are in bold. Chr: chromosome.

**Table 3:** Prioritizer analysis of complex-autism patient CNVs.

Gene Name	Chr	Gene start	Gene end	P-Value
GK5	3	143,365,112	143,427,107	0.016793
OVOS2	12	31,155,854	31,245,435	0.019992
TTC4	1	54,893,516	54,920,347	0.023590
METT10D	17	2,268,539	2,361,930	0.043182
ENK11	22	17,307,231	17,308,997	0.047181

The five most significant genes (nominal empiric P-Value<0.05) identified by topology-corrected Prioritizer analysis of 181 non-overlapping CNVs are given. Gene start and end positions based on NCBI V35 assembly. Chr: chromosome.

Gene-network analysis of the genes highlighted by Prioritizer in the non-complex-autism group revealed a putative gene-network in four CNVs containing genes known to operate in glycobiology (*B3GALT6*, *GCNT2*, *LARGE*, and *GALNT9*). Inspection of the remaining genes in the non-complex-autism group CNVs revealed three additional genes functioning in the same processes (*B4GALT1*, *ARSA*, and *GALNTL5*), for which Prioritizer had determined borderline significance (0.057<nominal p-value<0.074). These seven genes code for enzymes operating in different aspects of glycosylation. *B3GALT6*, *B4GALT1*, and *GCNT2* participate in N-linked protein glycosylation, while *LARGE*, *GALNT9*, and *GALNTL5* are involved in O-linked protein glycosylation. *ARSA* functions as one of the main enzymes in lipid glycosylation. In only one of the healthy controls a heterozygous loss at chromosome 6p24.3-p24.2, containing the first two non-coding exons of transcript variant 1of *GCNT2* (NM_145649), was identified. The six remaining glycobiology genes were not affected by copy-number changes in the control cohort.

### CNV validation and segregation analysis

For the seven CNVs that harbor the seven glycobiology-related genes (Table 4), we performed real-time quantitative PCR (qPCR) analysis with region-specific primer pairs on the DNA of patients and their parents, and on two unrelated controls (for primer sequences see Table S4). The qPCR analysis confirmed the CNV calls for these seven regions in the patients (four gains, three hemizygous losses). Genotyping of the parents showed that one CNV was of paternal origin, three were maternally inherited, and three had arisen *de novo* in the patient (Table 4).

**Table 4:** CNVs containing genes related to glycobiology in non-complex-autism patients.

Subject	Sex	Type	Chr.	CNV Start	CNV End	Size (kb)	Genes	Origin
138-003	M	Gain	22q12.3	31,759,390	32,408,541	649.15	SYN3, **LARGE**	Paternal
021-003	M	Loss	6p24.3–p24.2	10,596,587	10,664,858	68.27	**GCNT2**	Maternal
104-003	M	Gain	9p21.1–p13.3	33,082,835	33,262,424	179.59	**B4GALT1,** SPINK4, BAG1, CHMP5	Maternal
088-004	F	Gain	7q36.1	150,928,784	151,150,935	212.15	PRKAG2, **GALNTL5**	Maternal
140-003	F	Gain	22q13.33	49,353,082	49,368,395	15.31	**ARSA**	*De novo*
143-003	M	Loss	12q24.33	131,285,329	131,370,031	84.70	DDX51, NOC4L, **GALNT9**	*De novo*
027-003	M	Loss	1p36.33	1,192,554	1,232,438	39.90	SDF4, **B3GALT6,** FAM132A, UBE2J2	*De novo*

CNV start and end positions based on NCBI V35 assembly. Genes related to glycobiology in bold. M: male, F: female.

### Gene expression analysis during murine brain development (figure 1 about here)

**Figure 1:**
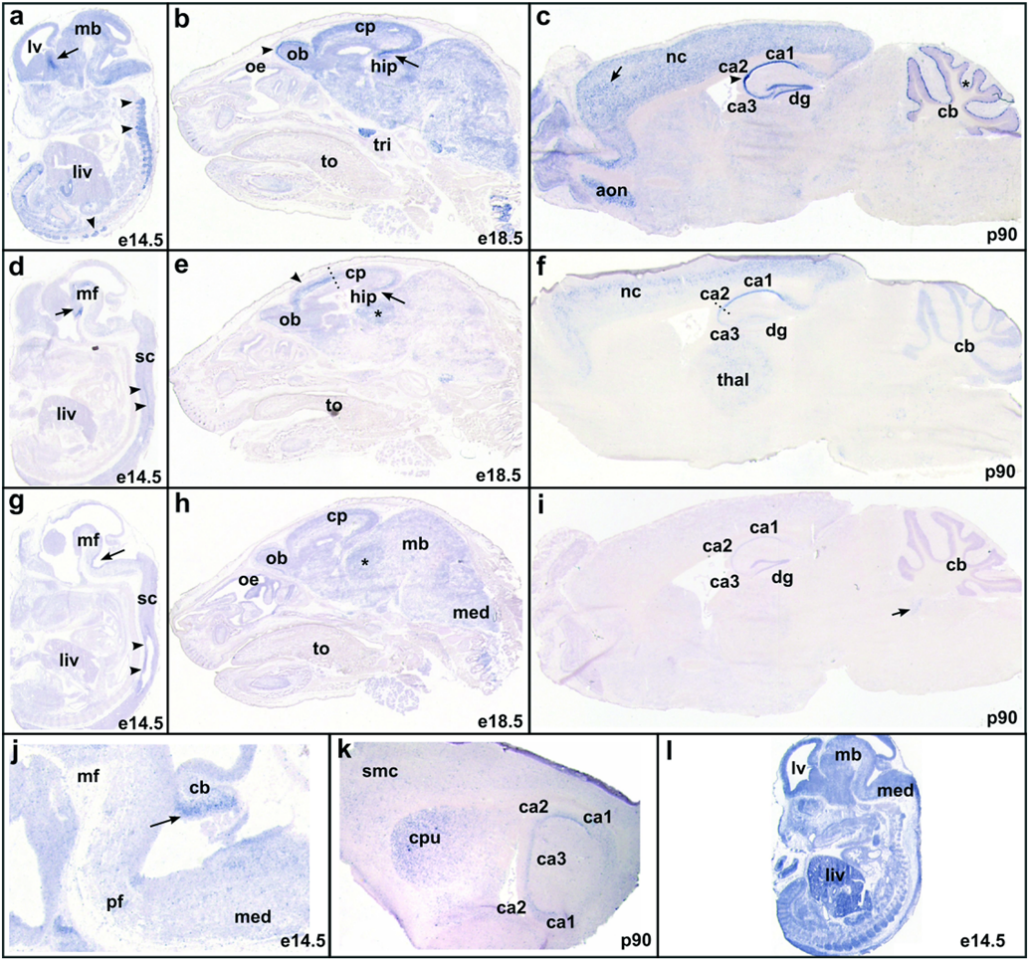
Expression analysis during mouse brain development of glycobiology-related genes overrepresented in autism-associated CNVs. a–c: mLarge, d–f: mGalnt9, g–i: mArsa, j,k: mGcnt2, l: mB3galt6. a) At E14.5 mLarge is expressed throughout the nervous system. The expression in the interior of the medial ganglionic eminence (MGE, (arrow), and in the dorsal root ganglia (DRG's, arrowheads) is higher then in surrounding tissues. b) At E18.5 expression is maintained in the nervous system, more intense expression was observed in the cortical plate and the developing cornu ammonis (CA) regions of the hippocampus (arrow), and in the mitral cells of the olfactory bulb (arrowhead). c) In the adult brain mLarge expression is high in the cerebral cortex and hippocampus. The anterior cerebral cortex (arrow) and the CA2 region of the hippocampus (arrowhead) show more intense staining than adjacent cells. In the cerebellum mLARGE is highly expressed in the Purkinje cell layer (asterisk). d) A low level of mGalnt9 expression was observed in the cortical plate at E14.5, stronger staining was present in the region of the future hypothalamus (arrow), and in a stripe of cells adjacent to the central canal of the spinal cord (arrowheads). e) At E18.5 neural expression of mGalnt9 has become restricted to the forebrain, with the exception of the thalamus (asterisk), and some hindbrain nuclei (not shown). The arrowhead and arrow indicate higher levels of mGalnt9 expression in the anterior cortical plate, and in the hippocampus, respectively. The transition between high and low level expression in the cortical plate is marked by a dotted line. f) In the adult brain mGalnt9 was expressed in layer II/III pyramidal neurons of the cerebral cortex, and in the CA1 and CA2 region of the hippocampus. Expression at a lower level was present in the thalamus and in the Purkinje and granular cell layer of the cerebellum. g) At E14.5 mArsa expression was observed in the floorplate neuroepithelium of the midbrain (arrow), extending caudally into the spinal cord (arrowheads). A low intensity signal was also present in the thalamus. h) At E18.5 expression of mArsa was maintained throughout the nervous system, with highest expression in the cortical plate, and thalamus (asterisk). i) In adult brain a low level of mArsa expression was observed in the CA and DG regions of the hippocampus, and in the granular cell layer of the cerebellum. Additionally, the medial vestibular nucleus showed expression of mArsa. j) Expression of mGcnt2 in the cerebellar anlage (arrow) at E14.5. k) In the adult brain, mGcnt2 was expressed in the CA regions of the hippocampus, and in the lateral portion of the caudate putamen. l) mB3galt6 was ubiquitously expressed from E14.5 onwards. Magnifications: 12.5×, except j: 25×. Abbreviations: aon: anterior olfactory nucleus, cb: cerebellum, cp: cortical plate, cpu: caudate putamen, dg: dentate gyrus, hip: hippocampus, liv: liver, lv: lateral ventricle, mb: midbrain, med: medulla oblongata, mf: mesencephalic flexure, nc: neocortex, ob: olfactory bulb, oe: olfactory epithelium, pf: pontine flexure, thal: thalamus, to: tongue, tri: trigeminal ganglion, sc: spinal cord, smc: primary somatosensory cortex.

Our finding that enzymes functioning in glycobiology were overrepresented in CNVs in multiple unrelated non-complex-autism patients may point towards a role for these enzymes in brain development. As Prioritizer does not use information regarding the tissues in which genes are expressed, we performed an independent expression analysis using mouse transcript-specific cRNA *in situ* hybridization for the seven glycobiology-related genes on murine embryonic and postnatal brain sections, in order to determine if, when, and where these genes were expressed in the developing brain (Table S3 and Fig. 1). Transcripts of three of the candidate genes, *B3GALT6*, *B4GALT1*, and *GALNTL5*, were ubiquitously expressed throughout the murine embryo, including the nervous system (Fig. 1, and not shown). More restricted and specific expression patterns during brain development were observed for transcripts of *LARGE*, *ARSA*, *GCNT2* and *GALNT9*. Specifically, *LARGE* transcripts were detected in the embryonic as well as the adult murine cerebral cortex, and hippocampus. A slightly more robust expression was observed in the anterior cerebral cortex in adult brain. Additionally, the Purkinje cells of the cerebellum showed expression of *LARGE* in the adult brain. *GALNT9* was expressed in the cortical plate and future hypothalamus of the embryonic brain. Staining in the anterior cerebral cortex at E18.5 was more intense, suggestion higher expression levels in this domain. In the adult brain highest expression levels of *GALNT9* were observed in the cornu ammonis (CA)1- and CA2-regions of the hippocampus, and layer2/3 pyramidal neurons in the cerebral cortex. Low levels of staining were observed in the thalamus and granular cell layer of the cerebellum. *ARSA* transcripts were detected in the floorplate neuroepithelium from the midbrain caudally into the spinal cord at E14.5. At E18.5 staining was observed throughout the cortical plate, in the mitral cells of the olfactory bulb, and in the thalamus. In the adult brain, only low intensity staining was present in the hippocampus, and in the region of the medial vestibular nucleus. The mitral cells of the olfactory bulb maintained *ARSA* expression. The most prominent expression of *GCNT2* in the embryonic brain was in the granular cells of the cerebellum, and the floorplate neuroepithelium posterior to the midbrain. In the adult mouse brain staining was present in the CA-regions of the hippocampus, and in the lateral region of the caudate putamen.

### Prioritizer analysis of literature derived autism susceptibility regions

In order to see whether glycobiology-related genes are commonly present in regions that confer risk for autism, we performed an independent Prioritizer analysis of autism susceptibility loci derived from the literature. We recently published an overview of cytogenetic regions that revealed recurrent aberrations in autistic individuals [4]. Based on this study we identified seven cytogenetic regions that each had been identified in more than five autism cases that showed no chromosomal mosaicism (Table 5). Additionally, we selected six susceptibility loci that had been identified in multiple genetic linkage analysis studies (Table 5). Using Prioritizer, we investigated whether these 13 loci contained genes with functional relationship to each other, and whether these genes exert functions comparable to those of the glycobiology-related genes identified in the Prioritizer analysis of our microarray-based CNV screen. The top-ten ranked genes from each locus, from the topology-corrected Prioritizer analysis, are shown in Table 6. Interestingly, six genes involved in glycobiology were ranked highly in these loci (median ranked at 8^th^ position). These genes included *B3GALNT2* (1q42.2, ranked 8^th^ out of 138 genes, P = 0.06), *B3GALT1* (2q31.1, ranked 10^th^ out of 109 genes, P = 0.09), *GAL3ST2* (2q37, ranked 3^rd^ out of 77 genes, P = 0.06), *B3GNT5* (3q26.32, ranked 5^th^ out of 120 genes, P = 0.06), *GALNTL5* (7q36, ranked 9^th^ out of 105 genes, P = 0.10) and *ARSA* (22q13.3, ranked 8^th^ out of 51 genes, P = 0.26), of which the latter two were also prioritized in the CNVs of the non-complex-autism group. Although individual determined empiric P-Values for these glycobiology genes were not significant, these results are suggestive that analysis of loci previously implicated in autism also ranks genes functioning in glycobiology high.

**Table 5:** Literature derived autism susceptibility loci.

Locus	Chromosomal Location	No of Genes	Reference
1q42.2 (D1S1656)	217,212,087–237,212,087	**138**	[30]
2q31.1 (D2S2188)	165,430,238–185,430,238	**109**	[31]
2q37	233,875,000–243,020,000	**77**	[4]
3q26.32 (D3S3037)	168,924,373–188,924,373	**120**	[32]
5p15	0–16,900,000	**65**	[4]
7q22.1 (D7S477)	90,370,231–110,370,231	**193**	[31]
7q36.2 (D7S2462)	142,999,403–162,999,403	**105**	[32]
15q11–14	18,940,000–31,390,000	**65**	[4]
17q11.2 (D17S1294)	15,406,471–35,406,471	**272**	[33]
18q21–23	41,800,000–73,160,000	**117**	[4]
22q11.2	16,970,000–20,830,000	**74**	[4]
22q13.3	44,555,000–49,550,000	**51**	[4]
Xp22	0–24,700,000	**122**	[4]
	Total number of genes	**1,508**	

For loci identified through linkage analysis, the microsatellite markers are given between brackets. For each locus (for definition see methods) the chromosomal location based on the NCBI V35 assembly is provided, including the number of genes contained in the locus.

**Table 6:** Prioritizer analysis of literature-derived autism susceptibility loci.

Locus	Top ten ranked genes from topology-corrected Prioritizer analysis.
1q42.2 (D1S1656)	TRIM11 (0.002), NP_115811.1 (0.007), EXOC8 (0.015), ERO1LB (0.015), FMN2 (0.036), HIST3H3 (0.046), DEGS1 (0.047), **B3GALNT2** (0.061), TRIM17 (0.066), SIPA1L2 (0.077)
2q31.1 (D2S2188)	LRP2 (0.007), SCN1A (0.012), CYBRD1 (0.015), GAD1 (0.018), MTX2 (0.026), DHRS9 (0.033), NOSTRIN (0.050), ENH1_HUMAN (0.063), SCN2A2 (0.082), **B3GALT1** (0.095)
2q37	SEPT2_HUMAN (0.003), NP_612209.1 (0.053), **GAL3ST2** (0.060), GPR35 (0.072), SH3BP4 (0.076), RAMP1 (0.121), GBX2 (0.174), MLPH (0.185), AGXT (0.222), BOK (0.268)
3q26.32 (D3S3037)	GAW2_HUMAN (0.008), NP_872343.2 (0.021), NLGN1 (0.042), SIAT1_HUMAN (0.048), **B3GNT5** (0.064), NM_003002.1 (0.069), DNAJB11 (0.100), TBL1XR1 (0.136), MYNN (0.142), PSMD2 (0.148)
5p15	SEC6L1 (0.007), NP_954584.1 (0.032), XP_059689.3 (0.046), DAP (0.143), SLC9A3 (0.165), SRD5A1 (0.187), MTRR (0.219), IRX1 (0.281), SLC12A7 (0.284), SLC6A3 (0.292)
7q22.1 (D7S477)	CYP3A5 (0.003), CYP3A7 (0.003), CYP3A43 (0.003), CYP3A4 (0.007), GJE1 (0.008), FZD1 (0.019), MCM7 (0.035), BCAP29 (0.056), KRIT1 (0.058), MLL5 (0.059)
7q36.2 (D7S2462)	EFC2_HUMAN (0.007), PDIA4 (0.016), NP_065178.1 (0.030), ABCB8 (0.046), SHH (0.059), RNF32 (0.065), DPP6 (0.092), GIMAP6 (0.096), **GALNTL5** (0.102), NOS3 (0.103)
15q11–14	MKRN3 (0.009), TUBGCP5 (0.011), O60374_HUMAN (0.031), UBE3A (0.051), NIPA2 (0.078), NP_001019852.1 (0.128), KLF13 (0.144), NP_061831.1 (0.161), NP_055782.2 (0.260), GREM1 (0.370)
17q11.2 (D17S1294)	ALDH3A1 (0.011), PRR6 (0.015), ALDH3A2 (0.020), Q6NXQ4_HUMAN (0.026), PIGW (0.030), ACCN1 (0.039), ZPBP2_HUMAN (0.040), PIGS (0.049), DUSP14 (0.050), PM1_HUMAN (0.057)
18q21–23	FECH (0.013), RAB27B (0.023), POLI (0.030), PIGN (0.039), KIAA0427 (0.044), MBP (0.106), ST8SIA5 (0.120), ZNF236 (0.123), CNDP1 (0.140), PHLPP_HUMAN (0.157)
22q11.2	SLC25A1 (0.004), SDF2L1 (0.019), ENK11_HUMAN (0.034), DGCR2 (0.073), GSCL (0.075), RTN4R (0.092), COMT (0.108), PPM1F (0.152), CLDN5 (0.177), SEPT5 (0.233)
22q13.3	TUBGCP6 (0.006), MLC1 (0.046), ALG12 (0.070), MAPK11 (0.113), ADM2 (0.125), CERK (0.128), MAPK8IP2 (0.213), **ARSA** (0.260), PLXNB2 (0.286), TBC1D22A (0.301)
Xp22	MID1 (0.005), PCYT1B (0.015), NLGN4X (0.015), HCCS (0.020), ASMT (0.034), Q7Z4W4_HUMAN (0.040), CNKSR2 (0.040), GLRA2 (0.068), PLCXD1 (0.095), PTCHD1 (0.120)

The ten most significant genes identified from topology-corrected Prioritizer analysis of the 13 non-overlapping literature-derived autism susceptibility loci are given per locus (sorted on P-Value). Genes related to glycobiology are in bold. P-values for individual genes are given between brackets.

## Discussion

Recently, a number of studies have highlighted the causal role genomic CNVs may play in the occurrence of autism and ASD [4], [5], [8], [9]. Whereas these studies showed that a large percentage of autism patients carry potentially harmful genomic gains or losses, remarkably few of these aberrations were found to be recurrent. The size of the CNVs and the high degree of genetic heterogeneity among patient cohorts are factors that have hampered identification of susceptibility genes within the autism-associated CNVs. Consequently, candidate gene selection has remained a highly biased process. An additional problem has been the high degree of variability in the clinical features and co-morbidities of individual autism patients used for these studies, which may obscure the identification of risk genes acting in a subgroup of patients.

Taking these matters into account and trying to minimize confounding effects, we set out to perform a high resolution, genome-wide CNV screen in a genetically homogeneous Dutch autism cohort, followed by subsequent candidate gene selection of the genes in these CNVs using a novel bioinformatics tool, Prioritizer. Furthermore, the genes in 13 literature-derived autism susceptibility loci, including linkage regions and cytogenetically relevant regions, were analyzed as cross reference for the gene analysis of our initial CNV data.

First, to obtain a more uniform patient cohort and limiting the variability in clinical features, focusing strictly on autism, we applied a slightly modified version of a clinical checklist to clinical data obtained from our patients (see methods). This resulted in the formation of two autism patient groups; non-complex-autism and complex-autism. The phenotype of the two groups differed in the number of abnormalities in family history, growth disorders, and dysmorphic features or congenital anomalies. The non-complex-autism phenotype group was characterized by little or none of the aforementioned abnormalities, the complex-autism phenotype group was typified by the presence of multiple abnormalities. Similar to the original assumption by de Vries et al.[16], we hypothesized that these phenotypical differences may reflect a difference in the genomic defects underlying the disorder. However, no significant difference in the number or size of CNVs was observed between these patient groups, suggesting that the genes within these regions are responsible for the difference in phenotypes, not the total number of genes that are affected by genomic gain or loss.

Second, we identified a number of plausible novel autism candidate genes from our CNV regions using Prioritizer [11]. The Prioritizer analysis of CNVs in the non-complex-autism group has yielded meaningful data, since several of the genes highly ranked have previously been associated with cognitive or neuropsychiatric disorders. *Retinoic acid induced 1* (*RAI1*), located in an unstable region on chromosome 17p11.2, is involved in Smith-Magenis syndrome (MIM182290) [17], [18], a disorder with cognitive impairment and behavioral abnormalities. *Bromodomain-containing protein 1* (*BRD1*), at chromosome 22q13, has recently been associated with schizophrenia and bipolar affective disorder [19]. The *LARGE* gene, at 22q12.3, has recently been implicated in Walker-Warburg syndrome [20], a rare autosomal recessive disorder with mental retardation and muscular dystrophy, and disruption of the *LARGE* gene was observed in a patient with schizophrenia [21]. Moreover, these neuropsychiatric disorders display several clinical features that overlap with autism. Many other genes with known or putative functions in neuronal development, axon growth, and synaptic function were ranked highly, including n*eurotrimin* (*NTM*), p*iccolo* (*PCLO*), *D4 zinc and double PHD fingers family 1* (*DPF1,* also called *NeuD4*) and *S100 calcium binding protein A5* (*S100A5*). These genes show highly restricted brain expression patterns (The Allen brain atlas, www.brain-map.org)[22], specifically in brain regions where morphological alterations in post-mortem brains of autism patients have been identified (e.g. cerebral cortex, cerebellum, and hippocampus). In addition, these observations strengthen findings from structural and functional Magnetic Resonance Imaging studies [23].

Third, gene-network analysis of susceptibility genes in the non-complex-autism group and in 13 literature-derived autism-susceptibility loci revealed an overrepresentation of genes related to glycobiology, and suggests that dosage alterations in these genes could contribute to the autism phenotype. Congenital disorders of glycosylation (CDGs) are genetic diseases caused by defects in the synthesis, metabolism or functions of glycans, impacting on N- or O-linked protein glycosylation as well as lipid glycosylation [24], [25]. Whereas CDGs almost invariably show autosomal recessive inheritance and very severe disease phenotypes, the effects of gene dosage changes, as observed in autism patients, may reflect the expression of less severe dysfunction of the pathways in which these genes operate. Pedigree analysis of the patients carrying gains and losses of the glycobiology-related genes is in good agreement with a recently reported model for the genetics of autism, postulating that autism is mainly caused by either *de novo* mutations with high penetrance in males, or by mutations that are inherited from an unaffected mother [10].Three of the seven CNVs occurred *de novo* in the patients, and the other four CNVs were inherited from apparently unaffected parents, mostly of maternal origin (3 out of 4). The inheritance pattern shows that the effects of the observed gene dosage changes may not be fully penetrant, and interaction with other factors may be required to produce an autism phenotype.

The seven glycobiology-related genes identified in CNVs in our autism cohort are expressed in developing murine brain regions known to be altered in the human autistic brain. The essential role of protein glycosylation for normal brain development has been demonstrated by the severe brain phenotypes in Walker-Warburg syndrome and Muscle-Eye-Brain disease. These syndromes are caused by protein O-mannosyltransferase deficiencies resulting in truncation of the O-proteoglycan core. Also brain abnormalities resulting from defects in protein N-glycosylation have been found, while *ARSA* (arylsulfatase A)-deficiency leads to motor and mental symptoms (see below). In the present study we encountered genomic losses and gains in genes encoding enzymes involved in all of these glycosylation pathways. O-glycosylation was represented by *LARGE* (dup), *GALNT5* (dup) and *GALNT9* (del). Interestingly, a schizophrenia patient with a disruption in the *LARGE* gene [26] and the autism patient in the present study both carry a intragenic gain that may result in an internal disruption of the *LARGE* gene. N-glycosylation was represented by *B3GALT6* (del), *B4GALT1* (dup) and *GCNT2* (del). Deficiencies of beta1,3- and beta1,4-galactosyltransferases, particularly *B3GALTL* and *B4GALT1*, lead to neuronal phenotypes: *B3GALTL* deficiency, either by bi-allelic truncating mutations or by combination of genomic loss and point mutation, causes Peters Plus syndrome (MIM261540), an autosomal recessive syndrome with multiple symptoms including psychomotor retardation [27]. Psychomotor retardation was also observed in a patient with *B4GALT1* deficiency caused by a homozygous truncating mutation [28], indicating that galactosyltransferases play a role in development of the brain. *GCNT2* gives rise to the developmental I antigen of which some mutations cause cataract (MIM110800). No brain phenotypes are known, but our expression analysis shows that *CGNT2* displays a distinct spatiotemporal expression pattern suggestive for a function during brain development. The *ARSA* gene encodes the lysosomal enzyme arylsulfatase A, involved in cerebroside metabolism. Homozygous or compound heterozygous *ARSA* mutations cause metachromatic leukodystrophy (MLD, MIM250100) that displays early, late and adult forms, all with neurological and neuropsychiatric symptoms. In this study we report a gain of *ARSA* that could result in a gain-of-function of ARSA.

The fact that genomic gains as well as losses in these pathways appear to contribute to autism suggests that the ratios of the enzymes encoded by these genes is tightly regulated in the brain, and that changes in stoichiometry may lead to aberrant sugar chains on their protein substrates. Therefore, it will be paramount to identify the protein targets of these glycobiology-related genes in the brain, and to study their function. This will further increase our insight in the mechanisms by which they influence brain development, and how they can lead to neuropsychiatric disorders when functionally impaired. Ultimately, new possibilities for the development of pharmacological intervention strategies in the treatment of autism may emerge.

## Materials and Methods

### Ethics Statement

All subjects included in the study gave written informed consent, and the local Medical Ethics Review Boards approved all procedures.

### Patient and control cohort selection for genome-wide CNV analysis

At the department of Child and Adolescent Psychiatry of the UMC Utrecht an extensive repository of peripheral blood, genomic DNA, and phenotypic data on autism patients has been collected over the past 2 decades for research purposes. For autism patients, multidisciplinary evaluation included the Autism Diagnostic Interview – Revised (ADI-R)+/−the Autism Diagnostic Observation Schedule generic (ADOS-G). Clinical diagnosis was established by an experienced clinician who studied medical records, developmental history and available diagnostic information. All autism patients had above cut-off score for autism on the ADI-R/ADOS-G and were diagnosed according DSM-IV(TR)-criteria. Exclusion followed if ADI-R cut off criteria were not met, or if medical illness was present. A priory, the patients carrying known genetic defects (e.g. FMR1 or TSC1/2 mutations, major cytogenetic abnormalities on karyogram) were excluded from this study. From the remaining cases, a total of 105 subjects were selected for this study. The patient cohort almost entirely consisted of patients with autism and Asperger syndrome. In collaboration with several clinical geneticists in our hospital we applied a modified checklist, that was originally designed to assess the probability of a phenotype being caused by chromosomal aberrations in patients with mental retardation [16], to our autism patients medical statuses and medical reports. In this way we aimed to investigate whether a difference in genetic susceptibility exists between patients with only autism and patients with autism and additional abnormalities, and if the use of this prescreening tool could be extended to psychiatric disorders. The criteria we used to subdivide the phenotype according to clinical features are; a positive family history of autism and/or mental retardation, prenatal onset growth retardation (birth weight less than p3, according to gestational age), postnatal growth abnormalities (height or head circumference below −2 SD or above +2 SD, according to age), facial dysmorphic features and non-facial dysmorphic features or congenital anomalies. Subdividing the 105 subjects according to these clinical features resulted in the formation of two patient groups; the first group of autism patients (non-complex-autism group, n = 53) contained subjects with no or little of the aforementioned abnormalities. The second group of autism patients (complex-autism group, n = 52) consisted of subjects with more of the aforementioned abnormalities Additionally, 267 ethnically matched, unrelated, healthy volunteers (recruited by the Department of Neurology in the University Medical Center Utrecht) were included in this study. All controls were of self-declared Dutch descent with all four grandparents originating from The Netherlands.

### Autism susceptibility loci from genome-wide CNV analysis

Genomic DNA was isolated with a salting out procedure and genotyped using HumanHap300 Genotyping Beadchips (Illumina) as described [29]. All procedures were performed according to the manufacturer's protocol; 750 ng of genomic DNA was amplified, fragmented and hybridized to the array. Products were then fluorescently labeled and scanned using the Illumina Beadstation scanner. Raw data was uploaded in Beadstudio V2.3.41 genotyping software (Illumina) for further analysis. At the time of analysis of this study no automated CNV detection method was available for HumanHap300 Genotyping Beadchip data; therefore we formulated a script to detect three chromosomal aberration types from the Beadstudio analysis results: heterozygous deletions, homozygous deletions, and duplications (for detailed method see [15]). In short, heterozygous deletions were called when a series of SNPs showed absence of heterozygous genotypes (loss of heterozygosity, due to A/- or B/- genotypes) with negative deflections in the log_2_ R ratio. Homozygous deletions were defined as series of SNPs with strongly negative intensity signals (log_2_ R ratio<−3.0). Duplications were defined as series of SNPs with increased log_2_ R ratios, in the presence of B allele frequency values of ∼0·33 (AA/B genotypes) or ∼0·66 (A/BB genotypes).

### Definition of literature derived autism susceptibility loci

In this study, the literature derived loci for autism were based on data from four linkage studies [30]–[33] with multipoint logarithm of the odds score (MLS) above 3.0 [34], and the collected data from a large volume of cytogenetic studies [4]. Boundaries of the linkage regions were defined by a 20 MB basepair block centered around the most significantly linked marked in each locus. In these linkage based studies, cases with known cytogenetic aberrations have been excluded. Definition of the Cytogenetic Regions Of Interest (CROIs) was based on criteria which have been described before [4]: In short, regions on the human genome where multiple overlapping cytogenetic abnormalities co-occurred with an autism phenotype were identified through an extensive literature search. Cases involving chromosomal mosaicism or well described gene mutation as the most likely genetic cause for autism were excluded (e.g. patients with fragile-X syndrome caused by *FMR1* mutations). Accurate base pair positions of the linkage regions and CROIs were derived by mapping all available probe and/or banding information from the case reports to the NCBI V35 assembly. In the present study, only CROIs that contained more than 5 overlapping cases were selected for the analysis. In total 13 loci were defined of which six were based on linkage data and seven were based on cytogenetic data.

### Prioritization of positional autism candidate genes

To assess whether certain biological pathways were overrepresented with genes, mapping within the CNV regions that had been identified in the autistic individuals we used Prioritizer [11]. Prioritizer uses a reconstructed functional human gene network to prioritize genes residing in multiple loci, by assuming that real causal genes are more closely related within the human gene network than other genes. In order to establish accurate empiric P-values we used a permutation based, topology-corrected method. This permutation strategy assumes that in each permutation, the loci can be randomly shuffled across the genome. However, as we had only used the SNPs that are present on the Illumina HumanHap300 platform to identify CNVs, random shuffling of these loci will introduce a bias. To overcome this, we adapted Prioritizer's permutation procedure: We first determined what relationships exist between the genes that map within the CNVs identified either in non-complex-autism patients or in complex-autism patients. Subsequently, in each permutation we sampled an equal amount of CNVs from a set of CNVs that comprised both the 267 controls and either the non-complex or or complex-autism groups, respectively. While the inclusion of these patients in the permutation results in some loss of statistical power to detect significantly overrepresented pathways, this strategy ensures that all permuted loci reflect CNVs that have at least been identified once, resulting in a null-distribution that is not biased (data not shown).

### Validation and inheritance analysis of CNVs in autism pedigrees

Real-time quantitative PCR was performed on 40 ng of genomic DNA from the patients, both parents and 2 non-related controls (one male and one female) for the genes functioning in protein and lipid glycosylation. Region specific primer pairs for each CNV were designed using primer3 [35](Table S4). Reactions were carried out in duplo in a LightCycler 2.0 Real-Time PCR System (Roche Applied Science), using Lightcycler® Faststart DNA Master^PLUS^ SYBR Green I (Roche Applied science) according to the manufacturers protocols. Calculation of relative DNA quantities was performed using qBASE analyzer, version 1.3.5 [36].

### RNA *in situ* hybridization during mouse brain development

A detailed description of this method was reported previously [37]. In short, transcript specific sequences for the genes involved in protein glycosylation were amplified from embryonic day (E) 14.5 mouse brain total RNA using transcript specific primer sequences (Table S5) and the OneStep RT-PCR kit (Qiagen). The RT-PCR products were subsequently cloned into the pGEM-T-easy vector (Invitrogen), and DIG-labeled cRNA probes were made using T7 or Sp6 dependent RNA polymerase (Roche Applied Science). Sense and antisense probes were hybridized to 16 µm sagital cryosections of various mouse embryonic stages (E14.5, E16.5 and E18.5) and adult mouse brain, and expression was visualized using NBT-BCIP (Roche Applied Science). Images were recorded on a Zeiss Axioskop2 Plus microscope with a Sony Power HAD DXC-950P 3CCD color video camera. Expression was considered genuine only when sections hybridized with the corresponding sense probe showed no significant staining.

## Supporting Information

Table S1 Prioritizer input for the non-complex-autism patient group. The 210 CNVs identified in 53 patients with non-complex-autism were combined into 173 non-overlapping unique copy-number variant regions (CNVR) for analysis. Nucleotide positions for the CNVR start and end were based on the NCBI V35 assembly. Chr: chromosome.
(0.23 MB DOC)

Table S2 Prioritizer input for the complex-autism patient group. The 242 CNVs identified in 52 patients with a complex-autism phenotype were combined into 181 non-overlapping unique CNVR for analysis. Nucleotide positions for the CNVR start and end were based on the NCBI V35 assembly. Chr: chromosome.
(0.25 MB DOC)

Table S3 Summary of expression analysis by RNA in situ hybridization during murine brain development of glycobiology-related genes associated with autism. Genes from de novo occurring copy-number changes are in bold. CNS: central nervous system, PNS: peripheral nervous system, MGE: medial ganglionic eminence, DG: dentate gyrus.
(0.03 MB DOC)

Table S4 Primer sequences for real-time quantitative PCR analysis on human genomic DNA. Primers were designed using Primer3 (see methods).
(0.03 MB DOC)

Table S5 Primers sequences for generation of cRNA in situ hybridization probes from mouse RNA. Primers were designed using Primer3 (see methods).
(0.03 MB DOC)

## References

[1] Bailey A, Lecouteur A, Gottesman I, Bolton P, Simonoff E (1995). Autism as a strongly genetic disorder - Evidence from a British twin study.. Psychological Medicine.

[2] Lecouteur A, Bailey A, Goode S, Pickles A, Robertson S (1996). A broader phenotype of autism: The clinical spectrum in twins.. Journal of Child Psychology and Psychiatry and Allied Disciplines.

[3] Freitag CM (2007). The genetics of autistic disorders and its clinical relevance: a review of the literature.. Molecular Psychiatry.

[4] Vorstman JA, Staal WG, van Daalen E, Van Engeland H, Hochstenbach PF (2006). Identification of novel autism candidate regions through analysis of reported cytogenetic abnormalities associated with autism.. Mol Psychiatry.

[5] Sebat J, Lakshmi B, Malhotra D, Troge J, Lese-Martin C (2007). Strong association of de novo copy number mutations with autism.. Science.

[6] Morrow EM, Yoo SY, Flavell SW, Kim TK, Lin Y (2008). Identifying autism loci and genes by tracing recent shared ancestry.. Science.

[7] Beckmann JS, Estivill X, Antonarakis SE (2007). Copy number variants and genetic traits: closer to the resolution of phenotypic to genotypic variability.. Nature Reviews Genetics.

[8] Szatmari P, Paterson AD, Zwaigenbaum L, Roberts W, Brian J (2007). Mapping autism risk loci using genetic linkage and chromosomal rearrangements.. Nat Genet.

[9] Marshall CR, Noor A, Vincent JB, Lionel AC, Feuk L (2008). Structural variation of chromosomes in autism spectrum disorder.. Am J Hum Genet.

[10] Zhao X, Leotta A, Kustanovich V, LaJonchere C, Geschwind DH (2007). A unified genetic theory for sporadic and inherited autism.. Proceedings of the National Academy of Sciences of the United States of America.

[11] Franke L, van Bakel H, Fokkens L, de Jong ED, Egmont-Petersen M (2006). Reconstruction of a functional human gene network, with an application for prioritizing positional candidate genes.. American Journal of Human Genetics.

[12] Adie EA, Adams RR, Evans KL, Porteous DJ, Pickard BS (2006). SUSPECTS: enabling fast and effective prioritization of positional candidates.. Bioinformatics.

[13] Chen J, Xu H, Aronow BJ, Jegga AG (2007). Improved human disease candidate gene prioritization using mouse phenotype.. Bmc Bioinformatics.

[14] Aerts S, Lambrechts D, Maity S, Van Loo P, Coessens B (2006). Gene prioritization through genomic data fusion.. Nature Biotechnology.

[15] Blauw HM, Veldink JH, van Es MA, van Vught PW, Saris CG (2008). Copy-number variation in sporadic amyotrophic lateral sclerosis: a genome-wide screen.. Lancet Neurol.

[16] de Vries BBA, White SM, Knight SJL, Regan R, Homfray T (2001). Clinical studies on submicroscopic subtelomeric rearrangements: a checklist.. Journal of Medical Genetics.

[17] Seranski P, Hoff C, Radelof U, Hennig S, Reinhardt R (2001). RAI1 is a novel polyglutamine encoding gene that is deleted in Smith-Magenis syndrome patients.. Gene.

[18] Seranski P, Heiss NS, Dhorne-Pollet S, Radelof U, Korn B (1999). Transcription mapping in a medulloblastoma breakpoint interval and Smith-Magenis syndrome candidate region: Identification of 53 transcriptional units and new candidate genes.. Genomics.

[19] Severinsen JE, Bjarkam CR, Kiaer-Larsen S, Olsen IM, Nielsen MM (2006). Evidence implicating BRD1 with brain development and susceptibility to both schizophrenia and bipolar affective disorder.. Molecular Psychiatry.

[20] van Reeuwijk J, Grewal PK, Salih MAM, de Bernabe DBV, McLaughlan JM (2007). Intragenic deletion in the LARGE gene causes Walker-Warburg syndrome.. Human Genetics.

[21] Walsh T, McClellan JM, McCarthy SE, Addington AM, Pierce SB (2008). Rare structural variants disrupt multiple genes in neurodevelopmental pathways in schizophrenia.. Science.

[22] Lein ES, Hawrylycz MJ, Ao N, Ayres M, Bensinger A (2007). Genome-wide atlas of gene expression in the adult mouse brain.. Nature.

[23] Sundaram SK, Kumar A, Makki MI, Behen ME, Chugani HT (2008). Diffusion Tensor Imaging of Frontal Lobe in Autism Spectrum Disorder.. Cereb Cortex.

[24] Jaeken J, Matthijs G (2007). Congenital disorders of glycosylation: A rapidly expanding disease family.. Annual Review of Genomics and Human Genetics.

[25] Freeze HH, Aebi M (2005). Altered glycan structures: the molecular basis of congenital disorders of glycosylation.. Current Opinion in Structural Biology.

[26] Walsh T, McClellan JM, McCarthy SE, Addington AM, Pierce SB (2008). Rare Structural Variants Disrupt Multiple Genes in Neurodevelopmental Pathways in Schizophrenia.. Science.

[27] Lesnik Oberstein SA, Kriek M, White SJ, Kalf ME, Szuhai K (2006). Peters Plus syndrome is caused by mutations in B3GALTL, a putative glycosyltransferase.. Am J Hum Genet.

[28] Hansske B, Thiel C, Lubke T, Hasilik M, Honing S (2002). Deficiency of UDP-galactose : N-acetylglucosamine beta-1,4-galactosyltransferase I causes the congenital disorder of glycosylation type IId.. Journal of Clinical Investigation.

[29] Peiffer DA, Le JM, Steemers FJ, Chang WH, Jenniges T (2006). High-resolution genomic profiling of chromosomal aberrations using Infinium whole-genome genotyping.. Genome Res.

[30] Buxbaum JD, Silverman J, Keddache M, Smith CJ, Hollander E (2004). Linkage analysis for autism in a subset families with obsessive-compulsive behaviors: Evidence for an autism susceptibility gene on chromosome 1 and further support for susceptibility genes on chromosome 6 and 19.. Molecular Psychiatry.

[31] Palferman S, Matthews N, Turner M, Moore J, Hervas A (2001). A genomewide screen for autism: Strong evidence for linkage to chromosomes 2q, 7q, and 16p.. American Journal of Human Genetics.

[32] Auranen M, Vanhala R, Varilo T, Ayers K, Kempas E (2002). A genomewide screen for autism-spectrum disorders: Evidence for a major susceptibility locus on chromosome 3q25–27.. American Journal of Human Genetics.

[33] McCauley JL, Olson LM, Dowd M, Amin T, Steele A (2004). Linkage and association analysis at the serotonin transporter (SLC6A4) locus in a rigid-compulsive subset of autism.. American Journal of Medical Genetics Part B-Neuropsychiatric Genetics.

[34] Lander E, Kruglyak L (1995). Genetic Dissection of Complex Traits - Guidelines for Interpreting and Reporting Linkage Results.. Nature Genetics.

[35] Rozen S, Skaletsky H, Krawetz S, Misener S (2000). Primer3 on the WWW for general users and for biologist programmers.. Bioinformatics Methods and Protocols: Methods in Molecular Biology.

[36] Hellemans J, Mortier G, De Paepe A, Speleman F, Vandesompele J (2007). qBase relative quantification framework and software for management and automated analysis of real-time quantitative PCR data.. Genome Biol.

[37] van der Zwaag B, Burbach JPH, Scharfe C, Oefner PJ, Brunner HG (2005). Identifying new candidate genes for hereditary facial paresis on chromosome 3q21–q22 by RNA in situ hybridization in mouse.. Genomics.

