# Supplementary material for: Gene-Network Analysis Identifies Susceptibility Genes Related to Glycobiology in Autism
Source: PLoS One. 2009 May 28;4(5):e5324. doi: 10.1371/journal.pone.0005324 (PMC2683930; doi:10.1371/journal.pone.0005324)
Supplement: Table S1 — Prioritizer input for the non-complex-autism patient group. The 210 CNVs identified in 53 patients with non-complex-autism were combined into 173 non-overlapping unique copy-number variant regions (CNVR) for analysis. Nucleotide positions for the CNVR start and end were based on the NCBI V35 assembly. Chr: chromosome. (0.23 MB DOC) [file pone.0005324.s001.doc]

**Table S1.** Prioritizer input for the non-complex-autism patient group. The 210 CNVs identified in 53 patients with non-complex-autism were combined into 173 non-overlapping unique copy-number variant regions (CNVR) for analysis. Nucleotide positions for the CNVR start and end were based on the NCBI V35 assembly. Chr: chromosome.

| Chr | Cytoband | CNVR Start | CNVR End | Size |
| --- | --- | --- | --- | --- |
| 1 | 1-p36.33 | 1198200 | 1232438 | 34239 |
| 1 | 1-p36.13 | 17339898 | 17367960 | 28063 |
| 1 | 1-p21.3 | 97750200 | 97912740 | 162541 |
| 1 | 1-p21.1 | 102379409 | 102497694 | 118286 |
| 1 | 1-p13.3 | 108411772 | 108592002 | 180231 |
| 1 | 1-q21.3 | 150175580 | 150322963 | 147384 |
| 1 | 1-q31.1 | 186130295 | 186267939 | 137645 |
| 1 | 1-q31.1-2 | 186492708 | 186550010 | 57303 |
| 1 | 1-q43 | 234979009 | 235103569 | 124561 |
| 2 | 2-p23.1 | 30403207 | 30415898 | 12692 |
| 2 | 2-p22.1 | 41014697 | 41111264 | 96568 |
| 2 | 2-p16.3 | 50970396 | 51006704 | 36309 |
| 2 | 2-p12 | 76852704 | 76859011 | 6308 |
| 2 | 2-p12-2 | 78576027 | 78755557 | 179531 |
| 2 | 2-p12-3 | 79321813 | 79399940 | 78128 |
| 2 | 2-p12-4 | 82137266 | 82230054 | 92789 |
| 2 | 2-p12-5 | 82804452 | 83032385 | 227934 |
| 2 | 2-q13 | 110214618 | 110292098 | 77481 |
| 2 | 2-q14.2 | 119290257 | 119334926 | 44670 |
| 2 | 2-q21.2 | 131998586 | 132218113 | 219528 |
| 2 | 2-q37.1 | 234721481 | 234725592 | 4112 |
| 3 | 3-p25.1 | 14281118 | 14340270 | 59153 |
| 3 | 3-p24.3 | 21234439 | 21320230 | 85792 |
| 3 | 3-p24.3-2 | 22540247 | 22583916 | 43670 |
| 3 | 3-p21.31 | 47724712 | 47746291 | 21580 |
| 3 | 3-p21.31-2 | 50177235 | 50230309 | 53075 |
| 3 | 3-p13 | 72676483 | 72765009 | 88527 |
| 3 | 3-p12.3 | 75038271 | 75281594 | 243324 |
| 3 | 3-p12.3-2 | 75511365 | 75650909 | 139545 |
| 3 | 3-p12.2 | 82281157 | 82362657 | 81501 |
| 3 | 3-p11.2 | 89906177 | 90391748 | 485572 |
| 3 | 3-q11.2 | 97815779 | 97982225 | 166447 |
| 3 | 3-q13.33 | 122406894 | 122529158 | 122265 |
| 3 | 3-q25.31 | 156959249 | 157008500 | 49252 |
| 4 | 4-q12 | 57905392 | 57939482 | 34091 |
| 4 | 4-q13.1 | 63362450 | 63976895 | 614446 |
| 4 | 4-q13.1-2 | 65213723 | 65551580 | 337858 |
| 4 | 4-q21.3 | 88125696 | 88220949 | 95254 |
| 4 | 4-q22.3 | 98068885 | 98400850 | 331966 |
| 4 | 4-q28.1 | 127091656 | 127170680 | 79025 |
| 4 | 4-q28.2 | 129880123 | 129942768 | 62646 |
| 4 | 4-q28.3 | 132303980 | 132705494 | 401515 |
| 4 | 4-q32.2 | 162292947 | 162364429 | 71483 |
| 4 | 4-q32.3 | 166235940 | 166282047 | 46108 |
| 4 | 4-q34.3 | 178351811 | 178520504 | 168694 |
| 4 | 4-q35.2 | 191060452 | 191117840 | 57389 |
| 5 | 5-p15.33 | 1063083 | 1166244 | 103162 |
| 5 | 5-p13.3 | 32142841 | 32171356 | 28516 |
| 5 | 5-p11 | 45948724 | 46111714 | 162991 |
| 5 | 5-q11.1 | 49618507 | 50240987 | 622481 |
| 5 | 5-q12.1 | 60177002 | 60475337 | 298336 |
| 5 | 5-q14.1 | 77425729 | 77634610 | 208882 |
| 5 | 5-q15 | 97074222 | 97121798 | 47577 |
| 5 | 5-q23.1 | 120440249 | 120480755 | 40507 |
| 5 | 5-q35.1 | 170371347 | 170531269 | 159923 |
| 5 | 5-q35.2 | 175492445 | 175528566 | 36122 |
| 6 | 6-p24.3 | 10596587 | 10664858 | 68272 |
| 6 | 6-p12.3 | 50649372 | 63128231 | 12478860 |
| 6 | 6-q12 | 65351159 | 65453784 | 102626 |
| 6 | 6-q12-2 | 67061239 | 67104015 | 42777 |
| 6 | 6-q22.1 | 115505852 | 115733640 | 227789 |
| 6 | 6-q22.31 | 124424451 | 124551026 | 126576 |
| 6 | 6-q25.3 | 155955219 | 157389295 | 1434077 |
| 6 | 6-q27 | 168154636 | 168412512 | 257877 |
| 7 | 7-p21.3 | 12471037 | 12564727 | 93691 |
| 7 | 7-p21.1 | 16026892 | 16116222 | 89331 |
| 7 | 7-p15.3 | 21075425 | 21077798 | 2374 |
| 7 | 7-q11.23 | 75830279 | 76154870 | 324592 |
| 7 | 7-q21.11 | 82349216 | 82441060 | 91845 |
| 7 | 7-q22.1 | 98045250 | 98325873 | 280624 |
| 7 | 7-q22.1-2 | 100561798 | 100720890 | 159093 |
| 7 | 7-q22.3 | 104339246 | 104480631 | 141386 |
| 7 | 7-q32.2 | 128968608 | 129038902 | 70295 |
| 7 | 7-q33 | 134524489 | 134596266 | 71778 |
| 7 | 7-q34 | 142273019 | 142398377 | 125359 |
| 7 | 7-q36.1 | 150928784 | 151140935 | 212152 |
| 8 | 8-p23.2 | 2569376 | 3248890 | 679515 |
| 8 | 8-p23.2-2 | 4080526 | 4098532 | 18007 |
| 8 | 8-p21.2 | 25579053 | 25628787 | 49735 |
| 8 | 8-q11.1 | 47229654 | 47292439 | 62786 |
| 8 | 8-q13.1q13.2 | 67649469 | 68204344 | 554876 |
| 8 | 8-q21.3 | 87346691 | 87431822 | 85132 |
| 8 | 8-q21.3-2 | 89617838 | 89842767 | 224930 |
| 8 | 8-q24.23 | 137747933 | 137948247 | 200315 |
| 8 | 8-q24.23-2 | 138771563 | 139351291 | 579729 |
| 9 | 9-p24.1 | 5296824 | 5325470 | 28647 |
| 9 | 9-p23 | 9791907 | 9814382 | 22476 |
| 9 | 9-p23-2 | 10287519 | 10308490 | 20972 |
| 9 | 9-p23-3 | 11837376 | 11994095 | 156720 |
| 9 | 9-p21.3 | 24136203 | 24160867 | 24665 |
| 9 | 9-p21.1 | 28188623 | 28332179 | 143557 |
| 9 | 9-p21.1-2 | 28650665 | 28697299 | 46635 |
| 9 | 9-p21.1p13.3 | 33082835 | 33262424 | 179590 |
| 9 | 9-q21.11 | 69265923 | 69285951 | 20029 |
| 10 | 10-p15.3 | 742193 | 777896 | 35704 |
| 10 | 10-p15.2 | 3167320 | 3209445 | 42126 |
| 10 | 10-q11.22 | 47013328 | 47173619 | 160292 |
| 10 | 10-q21.3 | 67749354 | 67785209 | 35856 |
| 10 | 10-q26.3 | 135155270 | 135266329 | 111060 |
| 11 | 11-p15.4 | 5858528 | 5911385 | 52858 |
| 11 | 11-p13 | 34599069 | 34737512 | 138444 |
| 11 | 11-q22.3 | 107166452 | 107175438 | 8987 |
| 11 | 11-q25 | 131533491 | 131833030 | 299540 |
| 11 | 11-q25-2 | 134076145 | 134117064 | 40920 |
| 12 | 12-p13.31 | 7876208 | 8014573 | 138366 |
| 12 | 12-p12.3 | 19360345 | 19475400 | 115056 |
| 12 | 12-p11.21 | 31248369 | 31298174 | 49806 |
| 12 | 12-p11.21-2 | 31898694 | 31954269 | 55576 |
| 12 | 12-q13.13 | 50977370 | 51074279 | 96910 |
| 12 | 12-q14.1 | 56403912 | 56582765 | 178854 |
| 12 | 12-q14.1-2 | 58725952 | 58756300 | 30349 |
| 12 | 12-q21.1 | 73481976 | 73704544 | 222569 |
| 12 | 12-q21.31 | 81671084 | 81707620 | 36537 |
| 12 | 12-q24.33 | 130255197 | 130339814 | 84618 |
| 12 | 12-q24.33-2 | 131285329 | 131370031 | 84703 |
| 13 | 13-q13.3 | 34712496 | 34934119 | 221624 |
| 13 | 13-q14.3 | 52302730 | 52713799 | 411070 |
| 13 | 13-q33.2 | 105206201 | 105279569 | 73369 |
| 14 | 14-q11.2 | 21681307 | 21811574 | 130268 |
| 14 | 14-q13.1 | 33948776 | 34023130 | 74355 |
| 14 | 14-q21.1 | 37255412 | 37343673 | 88262 |
| 14 | 14-q21.3 | 46744261 | 46747936 | 3676 |
| 14 | 14-q23.2 | 61704333 | 61858700 | 154368 |
| 15 | 15-q11.2 | 19852603 | 19869474 | 16872 |
| 15 | 15-q11.2-2 | 20306549 | 20777695 | 471147 |
| 15 | 15-q13.2 | 28723577 | 28853522 | 129946 |
| 15 | 15-q14 | 31964180 | 32034914 | 70735 |
| 15 | 15-q14-2 | 32724681 | 32769236 | 44556 |
| 15 | 15-q21.1 | 43181698 | 43261663 | 79966 |
| 15 | 15-q21.3 | 54877059 | 55065361 | 188303 |
| 15 | 15-q24.3 | 74820989 | 74963213 | 142225 |
| 15 | 15-q25.3 | 85631534 | 85663813 | 32280 |
| 15 | 15-q26.3 | 98052198 | 98147574 | 95377 |
| 15 | 15-q26.3-2 | 99883984 | 100146047 | 262064 |
| 16 | 16-p11.2 | 30811180 | 31255249 | 444070 |
| 16 | 16-p11.1 | 34373576 | 34618468 | 244893 |
| 16 | 16-q23.2 | 79634322 | 79716938 | 82617 |
| 17 | 17-p11.2 | 17463669 | 17655826 | 192158 |
| 17 | 17-p11.2-2 | 18796336 | 19211040 | 414705 |
| 17 | 17-q11.2 | 24399965 | 24570756 | 170792 |
| 17 | 17-q21.31 | 39098027 | 39145491 | 47465 |
| 18 | 18-q12.3 | 40698471 | 40714434 | 15964 |
| 18 | 18-q22.1 | 63883577 | 63959826 | 76250 |
| 18 | 18-q22.3 | 67394719 | 67432490 | 37772 |
| 19 | 19-p13.3 | 1075031 | 1126396 | 51366 |
| 19 | 19-p13.2 | 7056136 | 7173832 | 117697 |
| 19 | 19-p13.2-2 | 7326223 | 7444117 | 117895 |
| 19 | 19-p13.2-3 | 10307568 | 10450422 | 142855 |
| 19 | 19-p12 | 20356521 | 20528316 | 171796 |
| 19 | 19-q13.13-q13.2 | 43388894 | 43647423 | 258530 |
| 19 | 19-q13.2 | 47035366 | 47196345 | 160980 |
| 19 | 19-q13.32 | 52584487 | 52640040 | 55554 |
| 19 | 19-q13.32-2 | 53449420 | 53456533 | 7114 |
| 19 | 19-q13.42 | 59086910 | 59629281 | 542372 |
| 19 | 19-q13.42-2 | 59996120 | 60069820 | 73701 |
| 20 | 20-p12.1 | 14654616 | 14913823 | 259208 |
| 20 | 20-q11.21 | 30522023 | 30677308 | 155286 |
| 20 | 20-q13.12 | 43860825 | 43988093 | 127269 |
| 20 | 20-q13.2 | 52081775 | 52088118 | 6344 |
| 21 | 21-q11.2 | 13523286 | 14027356 | 504071 |
| 21 | 21-q22.3 | 43647907 | 43653486 | 5580 |
| 22 | 22-q11.21 | 17252341 | 17367682 | 115342 |
| 22 | 22-q11.23 | 23965182 | 24234365 | 269184 |
| 22 | 22-q12.1 | 27123990 | 27377557 | 253568 |
| 22 | 22-q12.3 | 31759390 | 32408541 | 649152 |
| 22 | 22-q13.2 | 40047364 | 40395293 | 347930 |
| 22 | 22-q13.32q13.33 | 47513278 | 48570538 | 1057261 |
| 22 | 22-q13.33 | 49353082 | 49368395 | 15314 |
| X | X-p22.31 | 6607042 | 6700608 | 93567 |
| X | X-p22.12 | 19508204 | 19984850 | 476647 |
| X | X-p11.3 | 44168651 | 44173031 | 4381 |
| X | X-q12 | 66135717 | 67168358 | 1032642 |
| X | X-q27.3 | 145298049 | 145745559 | 447511 |
