# Supplementary material for: Gene-Network Analysis Identifies Susceptibility Genes Related to Glycobiology in Autism
Source: PLoS One. 2009 May 28;4(5):e5324. doi: 10.1371/journal.pone.0005324 (PMC2683930; doi:10.1371/journal.pone.0005324)
Supplement: Table S2 — Prioritizer input for the complex-autism patient group. The 242 CNVs identified in 52 patients with a complex-autism phenotype were combined into 181 non-overlapping unique CNVR for analysis. Nucleotide positions for the CNVR start and end were based on the NCBI V35 assembly. Chr: chromosome. (0.25 MB DOC) [file pone.0005324.s002.doc]

**Table S2.** Prioritizer input for the complex-autism patient group. The 242 CNVs identified in 52 patients with a complex-autism phenotype were combined into 181 non-overlapping unique CNVR for analysis. Nucleotide positions for the CNVR start and end were based on the NCBI V35 assembly. Chr: chromosome.

| Chr. | Cytoband | CNVR Start | CNVR End | Size |
| --- | --- | --- | --- | --- |
| 1 | 1-p36.32 | 2949536 | 3036847 | 87312 |
| 1 | 1-p36.32-2 | 3195086 | 3222374 | 27289 |
| 1 | 1-p36.13 | 16103391 | 16138663 | 35273 |
| 1 | 1-p36.13-2 | 17339898 | 17359115 | 19218 |
| 1 | 1-p34.3 | 35995289 | 36130215 | 134927 |
| 1 | 1-p32.2 | 54851762 | 54990862 | 139101 |
| 1 | 1-p31.1 | 72733264 | 72887515 | 154252 |
| 1 | 1-p21.1 | 105033263 | 105077321 | 44059 |
| 1 | 1-p13.2 | 112801048 | 113314448 | 513401 |
| 1 | 1-p13.1 | 115119915 | 115208223 | 88309 |
| 1 | 1-q21.3 | 150425206 | 150536110 | 110905 |
| 1 | 1-q24.2 | 165650424 | 165659244 | 8821 |
| 1 | 1-q31.2 | 188198352 | 188233756 | 35405 |
| 1 | 1-q31.2-2 | 188421032 | 188537183 | 116152 |
| 1 | 1-q31.3 | 193569717 | 193673150 | 103434 |
| 1 | 1-q42.2 | 230053825 | 230072426 | 18602 |
| 2 | 2-p24.2 | 18247833 | 18261797 | 13965 |
| 2 | 2-p22.3 | 32545341 | 33240045 | 694705 |
| 2 | 2-p16.3 | 49426382 | 49449446 | 23065 |
| 2 | 2-p16.1 | 55417332 | 55464646 | 47315 |
| 2 | 2-p14p13.3 | 68464846 | 68535303 | 70458 |
| 2 | 2-p11.2 | 87342385 | 87615568 | 273184 |
| 2 | 2-q14.1 | 117443275 | 117627587 | 184313 |
| 2 | 2-q34 | 211321093 | 211831425 | 510333 |
| 2 | 2-q34-2 | 213012540 | 213015999 | 3460 |
| 2 | 2-q37.3 | 242633058 | 242712341 | 79284 |
| 3 | 3-p26.3 | 812481 | 1071770 | 259290 |
| 3 | 3-p14.2 | 60043770 | 60251762 | 207993 |
| 3 | 3-p14.1 | 63840987 | 63878799 | 37813 |
| 3 | 3-p12.3 | 75511365 | 75650909 | 139545 |
| 3 | 3-q13.13 | 111216560 | 111461438 | 244879 |
| 3 | 3-q13.2 | 113585830 | 113597852 | 12023 |
| 3 | 3-q23 | 143295457 | 143554630 | 259174 |
| 3 | 3-q24 | 149045001 | 149145158 | 100158 |
| 3 | 3-q25.1 | 152997288 | 153028739 | 31452 |
| 3 | 3-q26.1 | 163361415 | 163455199 | 93785 |
| 3 | 3-q26.32 | 180338478 | 180510515 | 172038 |
| 4 | 4-p16.1p15.33 | 10983938 | 11009579 | 25642 |
| 4 | 4-p14 | 38277505 | 38288222 | 10718 |
| 4 | 4-q13.1 | 63547200 | 63949252 | 402053 |
| 4 | 4-q13.1-2 | 64324804 | 64492558 | 167755 |
| 4 | 4-q13.1-3 | 65696748 | 65847657 | 150910 |
| 4 | 4-q13.2 | 68336964 | 68410603 | 73640 |
| 4 | 4-q28.3 | 134578714 | 134627014 | 48301 |
| 4 | 4-q28.3-2 | 136413767 | 136616093 | 202327 |
| 4 | 4-q31.3 | 153348660 | 153359267 | 10608 |
| 4 | 4-q32.2 | 162607571 | 162675591 | 68021 |
| 4 | 4-q32.3 | 167441180 | 167520907 | 79728 |
| 4 | 4-q34.1 | 176311220 | 176406631 | 95412 |
| 4 | 4-q35.2 | 189519948 | 189975862 | 455915 |
| 4 | 4-q35.2-2 | 191060452 | 191086285 | 25834 |
| 5 | 5-p15.33 | 362096 | 409381 | 47286 |
| 5 | 5-p15.33-2 | 2194556 | 2271025 | 76470 |
| 5 | 5-p15.2 | 10660797 | 10724040 | 63244 |
| 5 | 5-p15.2-2 | 12314596 | 12589870 | 275275 |
| 5 | 5-p14.1 | 26822770 | 26870193 | 47424 |
| 5 | 5-q12.1 | 59758639 | 59807480 | 48842 |
| 5 | 5-q13.3 | 74015867 | 74028637 | 12771 |
| 5 | 5-q15 | 97053242 | 97121798 | 68557 |
| 5 | 5-q23.1 | 121282047 | 121379525 | 97479 |
| 5 | 5-q23.3 | 130124159 | 130177152 | 52994 |
| 5 | 5-q35.1 | 170308754 | 170465477 | 156724 |
| 5 | 5-q35.2 | 176108989 | 176238284 | 129296 |
| 6 | 6-p25.3 | 120522 | 139090 | 18569 |
| 6 | 6-p25.3-2 | 156941 | 184373 | 27433 |
| 6 | 6-p21.1 | 45203141 | 45248739 | 45599 |
| 6 | 6-q11.1 | 62021131 | 62094417 | 73287 |
| 6 | 6-q12 | 64938963 | 65054792 | 115830 |
| 6 | 6-q12-2 | 67075448 | 67104015 | 28568 |
| 6 | 6-q14.1 | 77372507 | 77379207 | 6701 |
| 6 | 6-q14.1-2 | 80395807 | 80410202 | 14396 |
| 7 | 7-p22.1 | 6323734 | 6444736 | 121003 |
| 7 | 7-p21.3 | 8606924 | 8632856 | 25933 |
| 7 | 7-p15.2 | 26916997 | 27060776 | 143780 |
| 7 | 7-q11.21 | 61848856 | 61882439 | 33584 |
| 7 | 7-q11.23 | 75005490 | 75015019 | 9530 |
| 7 | 7-q22.1 | 100561798 | 100720890 | 159093 |
| 7 | 7-q31.1 | 110754307 | 110840902 | 86596 |
| 7 | 7-q31.31 | 118528415 | 118569444 | 41030 |
| 7 | 7-q32.1 | 128066932 | 128097494 | 30563 |
| 7 | 7-q36.1 | 151336351 | 151495789 | 159439 |
| 7 | 7-q36.3 | 156616226 | 156633784 | 17559 |
| 7 | 7-q36.3-2 | 158410131 | 158618962 | 208832 |
| 8 | 8-p23.2p23.1 | 6117823 | 6301894 | 184072 |
| 8 | 8-p22 | 12943177 | 12954488 | 11312 |
| 8 | 8-p21.3 | 19160515 | 19203362 | 42848 |
| 8 | 8-p21.3-2 | 20728757 | 21312146 | 583390 |
| 8 | 8-p12 | 34774314 | 34829660 | 55347 |
| 8 | 8-q11.1 | 47647579 | 47654762 | 7184 |
| 8 | 8-q21.11 | 77081335 | 77144822 | 63488 |
| 8 | 8-q23.2 | 111771286 | 111791645 | 20360 |
| 8 | 8-q24.23 | 137757412 | 137919630 | 162219 |
| 9 | 9-p24.3 | 411860 | 794436 | 382577 |
| 9 | 9-p24.3-2 | 1721997 | 1749811 | 27815 |
| 9 | 9-p24.1 | 5296824 | 5335673 | 38850 |
| 9 | 9-p23 | 10651370 | 10676202 | 24833 |
| 9 | 9-p23-2 | 11631257 | 11641144 | 9888 |
| 9 | 9-p23-3 | 11993252 | 12144939 | 151688 |
| 9 | 9-p22.2 | 16893446 | 16908196 | 14751 |
| 9 | 9-p21.1 | 28344327 | 28372153 | 27827 |
| 9 | 9-p21.1-2 | 30433903 | 30548222 | 114320 |
| 9 | 9-q21.13 | 73677233 | 73855270 | 178038 |
| 9 | 9-q34.3 | 135360294 | 135540200 | 179907 |
| 10 | 10-p13 | 16022233 | 16101794 | 79562 |
| 10 | 10-p11.22 | 31695774 | 31848209 | 152436 |
| 10 | 10-q11.1 | 41756307 | 41837066 | 80760 |
| 10 | 10-q11.21 | 44582497 | 44626389 | 43893 |
| 10 | 10-q11.22 | 47013328 | 47173619 | 160292 |
| 10 | 10-q21.1 | 57079701 | 57174588 | 94888 |
| 10 | 10-q26.2 | 129791383 | 129792148 | 766 |
| 11 | 11-p15.4 | 3198799 | 3199943 | 1145 |
| 11 | 11-p14.2 | 26565073 | 26580062 | 14990 |
| 11 | 11-p12 | 37269530 | 37418196 | 148667 |
| 11 | 11-p12-2 | 38208761 | 38306184 | 97424 |
| 11 | 11-p11.12 | 50343409 | 51285867 | 942459 |
| 11 | 11-q22.1 | 99126002 | 99792469 | 666468 |
| 11 | 11-q22.3 | 107166452 | 107175438 | 8987 |
| 11 | 11-q25 | 133859037 | 134225383 | 366347 |
| 12 | 12-p13.32 | 4359496 | 4364199 | 4704 |
| 12 | 12-p13.31 | 7891603 | 8014573 | 122971 |
| 12 | 12-p12.3 | 19360345 | 19477127 | 116783 |
| 12 | 12-p12.1 | 24835636 | 24850538 | 14903 |
| 12 | 12-p11.21 | 31019283 | 31053044 | 33762 |
| 12 | 12-p11.21-2 | 31132612 | 31303936 | 171325 |
| 12 | 12-q14.2 | 62374612 | 62415375 | 40764 |
| 12 | 12-q15 | 68669987 | 68691987 | 22001 |
| 12 | 12-q21.1 | 72642095 | 72713878 | 71784 |
| 12 | 12-q22 | 93232045 | 93285740 | 53696 |
| 12 | 12-q24.33 | 130255197 | 130339814 | 84618 |
| 13 | 13-q12.11 | 19008195 | 19236643 | 228449 |
| 13 | 13-q31.2 | 87252080 | 87297156 | 45077 |
| 14 | 14-q21.1 | 37605924 | 37856371 | 250448 |
| 14 | 14-q21.2 | 44255590 | 44278526 | 22937 |
| 14 | 14-q21.3 | 47866053 | 47973238 | 107186 |
| 14 | 14-q23.2 | 61825486 | 62214839 | 389354 |
| 14 | 14-q31.3 | 85518391 | 85557882 | 39492 |
| 14 | 14-q32.2 | 98248060 | 98262193 | 14134 |
| 15 | 15-q11.2 | 20322358 | 20635884 | 313527 |
| 15 | 15-q13.2 | 28723577 | 28853522 | 129946 |
| 15 | 15-q13.3 | 30298847 | 30302218 | 3372 |
| 15 | 15-q22.2 | 58339218 | 58409103 | 69886 |
| 15 | 15-q26.1 | 91943094 | 91963332 | 20239 |
| 15 | 15-q26.3 | 99862888 | 100089798 | 226911 |
| 16 | 16-p13.2p13.13 | 10296913 | 10307787 | 10875 |
| 16 | 16-p11.2 | 30010303 | 30085308 | 75006 |
| 16 | 16-q22.1 | 67222756 | 67264434 | 41679 |
| 16 | 16-q23.1 | 76498035 | 76505221 | 7187 |
| 16 | 16-q23.1-2 | 76582490 | 76622640 | 40151 |
| 17 | 17-p13.3 | 1187693 | 1464664 | 276972 |
| 17 | 17-p13.3-2 | 2183154 | 2375258 | 192105 |
| 17 | 17-p11.2 | 21315172 | 21430683 | 115512 |
| 17 | 17-q21.33 | 45357976 | 45381048 | 23073 |
| 17 | 17-q24.3 | 66194150 | 66217618 | 23469 |
| 17 | 17-q25.3 | 74878104 | 74905197 | 27094 |
| 18 | 18-p11.32 | 1917798 | 1970668 | 52871 |
| 18 | 18-q22.1 | 64003719 | 64042401 | 38683 |
| 18 | 18-q22.1q22.2 | 64797539 | 64906488 | 108950 |
| 19 | 19-p13.3 | 1046716 | 1097100 | 50385 |
| 19 | 19-p13.2 | 9156591 | 9178125 | 21535 |
| 19 | 19-p12 | 20423788 | 20473895 | 50108 |
| 19 | 19-q13.2q13.31 | 48066441 | 48350666 | 284226 |
| 19 | 19-q13.31 | 48498399 | 48531928 | 33530 |
| 19 | 19-q13.42 | 59994795 | 60069820 | 75026 |
| 20 | 20-p12.3 | 8546485 | 8555393 | 8909 |
| 20 | 20-p12.1 | 14742361 | 14785965 | 43605 |
| 20 | 20-p12.1-2 | 14869606 | 15084730 | 215125 |
| 20 | 20-q13.2 | 52081775 | 52088118 | 6344 |
| 21 | 21-q11.2 | 13523286 | 13879844 | 356559 |
| 21 | 21-q22.3 | 43647907 | 43663581 | 15675 |
| 21 | 21-q22.3-2 | 44534535 | 44551218 | 16684 |
| 22 | 22-q11.1 | 15544076 | 15675771 | 131696 |
| 22 | 22-q11.21 | 17252341 | 20133739 | 2881399 |
| 22 | 22-q11.23 | 24012122 | 24235221 | 223100 |
| X | X-p22.2 | 11322903 | 11814229 | 491327 |
| X | X-p22.11 | 22443797 | 22454069 | 10273 |
| X | X-p21.1 | 33385479 | 33646053 | 260575 |
| X | X-p11.23 | 47594114 | 47605695 | 11582 |
| X | X-q21.33 | 97670499 | 97709224 | 38726 |
| X | X-q23 | 115724682 | 115798151 | 73470 |
| X | X-q26.3 | 134716598 | 134719525 | 2928 |
| X | X-q28 | 153491735 | 153642650 | 150916 |
