# Supplementary material for: Gene-Network Analysis Identifies Susceptibility Genes Related to Glycobiology in Autism
Source: PLoS One. 2009 May 28;4(5):e5324. doi: 10.1371/journal.pone.0005324 (PMC2683930; doi:10.1371/journal.pone.0005324)
Supplement: Table S3 — Summary of expression analysis by RNA in situ hybridization during murine brain development of glycobiology-related genes associated with autism. Genes from de novo occurring copy-number changes are in bold. CNS: central nervous system, PNS: peripheral nervous system, MGE: medial ganglionic eminence, DG: dentate gyrus. (0.03 MB DOC) [file pone.0005324.s003.doc]

**Table S3.** Summary of expression analysis by RNA *in situ* hybridization during murine brain development of glycobiology-related genes associated with autism. Genes from *de novo* occurring copy-number changes are in bold. CNS: central nervous system, PNS: peripheral nervous system, MGE: medial ganglionic eminence, DG: dentate gyrus.

| **Gene** | **E14.5** | **E18.5** | **Adult brain** |
| --- | --- | --- | --- |
| LARGE | Low level staining in the brain, more intense in mitral cell layer of the olfactory bulb, and cortical plate of the neocortex, including hippocampal anlage. Cells within the MGE stain positive, no signal in neuro-epithelium of MGE. Some nuclei in mid- and hindbrain more intensely stained. Also expressed in cranial and dorsal root ganglia. | Expression maintained throughout the brain, slightly darker staining in future CA regions of the hippocampus and in the cortical plate. Intense staining of mitral cell layer of the olfactory bulb. Neuronal cells of the retina also stained. Expression maintained in cranial and dorsal root ganglia. | Strong signal in pyramidal neurons throughout the cerebral cortex, most pronounced in prelimbic and frontal association cortex. Intense staining in neurons of the anterior olfactory nuclei. Pyramidal neurons of hippocampus intensely stained, especially cells in CA2 region, also staining in granular neurons of dentate gyrus. Purkinje cells of cerebellum intensely stained. |
| **GALNT9** | Expression in the cortical plate of the neocortex, in the ventral midbrain (around mesencephalic flexure) and in the future hypo-thalamus. In spinal cord expression in cells adjacent to the central canal. Low level staining in retinal neuronal cells. PNS staining in cranial and dorsal root ganglia. | Low intensity staining throughout the neocortex, staining more pronounced in (anterior) medial cerebral cortex and external granular layer of olfactory bulb. Now also staining observed in thalamus, and several nuclei in the hindbrain. Expression maintained in neuronal cell layer of the retina. | Intense staining in large neurons in cerebral cortex (layer 2/3) and CA1 and CA2 region of hippocampus, low level staining in neurons of CA3 region of hippocampus. Low intensity staining in Purkinje and granular cells of the cerebellum. Scattered cells stained throughout the thalamus, and in some small cell clusters in the hindbrain. |
| **ARSA** | Low intensity staining throughout the CNS, more intense in floor plate neuroepithelium of ventral mid- and hindbrain, and in thalamus. Staining also observed in cranial and dorsal root ganglia of PNS. | Expression maintained throughout CNS and PNS. Darker staining in the cortical plate of the neocortex, and in external granular and mitral cell layer of olfactory bulb. Staining also present in retinal neuronal cells and olfactory sensory neurons. | Low intensity staining maintained in pyramidal and granular neurons of hippocampus, and in the medial vestibular nucleus. Staining in the olfactory bulb maintained in mitral cell layer. |
| GCNT2 | Staining in the cerebellar anlage, and floor plate neuro-epithelium of the hindbrain and spinal cord. Olfactory sensory neurons also stained. Staining throughout PNS, including cranial and dorsal root ganglia. | Staining maintained in granular cell layer of the cerebellum, olfactory sensory neurons, and in ganglia of PNS. Low intensity signal in the floorplate neuro-epithelium of the forebrain. | Low level staining in the pyramidal and granular cells of the hippocampus, in the lateral striatum, and cerebral cortex. Cortical staining is most intense in anterior prelimbic and medial orbital region. |
| **B3GALT6** | Ubiquitous staining throughout the embryo. | Ubiquitous staining throughout the embryo. | Low intensity staining in pyramidal neurons of CA regions and granular neurons of DG of the hippocampus. |
| B4GALT1 | Ubiquitous staining throughout the nervous system. | Ubiquitous staining throughout the nervous system. | Ubiquitous staining throughout the nervous system. |
| GALNTL5 | Ubiquitous staining throughout the embryo. | Ubiquitous staining throughout the embryo. | Ubiquitous low level staining throughout the nervous system. |
