# Supplementary material for: Gene-Network Analysis Identifies Susceptibility Genes Related to Glycobiology in Autism
Source: PLoS One. 2009 May 28;4(5):e5324. doi: 10.1371/journal.pone.0005324 (PMC2683930; doi:10.1371/journal.pone.0005324)
Supplement: Table S4 — Primer sequences for real-time quantitative PCR analysis on human genomic DNA. Primers were designed using Primer3 (see methods). (0.03 MB DOC) [file pone.0005324.s004.doc]

**Table S4**: Primer sequences for real-time quantitative PCR analysis on human genomic DNA. Primers were designed using Primer3 (see methods).

| **CNV region** | **Forward primer** | **Reverse primer** |
| --- | --- | --- |
| 1p36.33 | GATAGCAAGACGCTACCTCA | GGTGACCAAAGTTTTCAACA |
| 6p24.3-p24.2 | gcttattcgtccaaggttgc | ttaaagcagggttgctcacc |
| 7q36.1 | GAAGTAGGAGAGAAAGAG | AGATGTGATTTTAGCAGCAG |
| 9p21.1-p13.3 | GACTTTGGCTGCTAGATGTG | AGAGTTACCCAAGTTTCTGACA |
| 12q24.33 | agagccgctccttgaatg | agcaccctgtaccagctctc |
| 22q12.3 | CTATGGATTGGGGTTAGGCT | GTGAAGACATGTTTGAGAGGC |
| 22q13.33 | gctgcttgccaagaacatag | gccccacacctctaagtcac |
