# Supplementary material for: Gene-Network Analysis Identifies Susceptibility Genes Related to Glycobiology in Autism
Source: PLoS One. 2009 May 28;4(5):e5324. doi: 10.1371/journal.pone.0005324 (PMC2683930; doi:10.1371/journal.pone.0005324)
Supplement: Table S5 — Primers sequences for generation of cRNA in situ hybridization probes from mouse RNA. Primers were designed using Primer3 (see methods). (0.03 MB DOC) [file pone.0005324.s005.doc]

**Table S5**: Primers sequences for generation of cRNA *in situ* hybridization probes from mouse RNA. Primers were designed using Primer3 (see methods).

| **Target gene** | **Forward primer** | **Reverse primer** |
| --- | --- | --- |
| B3galt6 | GGCACCACTCTGTTGTACCTG | CTGCAGTGACATCAGGGAAC |
| Gcnt2 | CACTACGTCCATGGCATTTG | GTGATGCTGGAGATGGAACC |
| Galntl5 | GTGCCAGATTCCAGGGATAA | CAGGCGTTTCCTTAATTCCA |
| B4galt1 | ATGTGGACCTCATTCCGATG | TCCCCAGGTCACAATTTAGC |
| Galnt9 | CCGACTCCAAGTGTCTGGTAG | AAGTGCACCGAGTCTCATCC |
| Large | TTTCCCCAAGTCAAAAGCAG | CCAGCTAGGTGGCAGGATAC |
| Arsa | CTTCTTCTACCCGCCCTACC | GAAAGCCTGTCTTCCAGGTG |
